# Kuakini HHP Center for Translational Research on Aging: An Introduction

**DOI:** 10.1093/geroni/igab046.1416

**Published:** 2021-12-17

**Authors:** Bradley Willcox, Kamal Masaki, Tim Donlon, Donald Willcox, Brian Morris, Richard Allsopp

**Affiliations:** 1 Kuakini Medical Center, Kuakini Medical Center/Honolulu, Hawaii, United States; 2 Kuakini Medical Center, Honolulu, Hawaii, United States; 3 JABSOM, Honolulu, Hawaii, United States; 4 Department Of Gerontology And Public Health, Okinawa International University, Okinawa, Japan; 5 University of Sydney, Sydney, New South Wales, Australia

## Abstract

Kuakini Medical Center (Kuakini) is establishing an interdisciplinary Hawai’i-based Center of Biomedical Research Excellence (COBRE) for clinical and translational research on aging. - Specific Aim 1: Create a world class, innovative, interdisciplinary Center for Translational Research on Aging by building on the existing rare and valuable Kuakini Honolulu Heart Program (Kuakini HHP) and its related studies and research infrastructure. - Specific Aim 2: Advance the genomic, biological and clinical science of aging, and enhance the world class character of our Center, by enlisting accomplished senior scientists to mentor promising Hawaii-based new investigators in interdisciplinary aging research and career development. - Specific Aim 3: Elevate the capacity of our Center by: i) creating a state-of-the-art Clinical and Translational Core, and ii) collaborating with other Centers of Biomedical Research Excellence. This presentation will briefly introduce the Center and discuss its aims, research strategy, and future plans.

